# Artificial Intelligence‐Augmented Morphometric Reporting for Standardized Facial Aesthetic Treatment Planning: A Multi‐Rater Reliability Study

**DOI:** 10.1111/jocd.70900

**Published:** 2026-05-17

**Authors:** Ömer Karakoyun, Kadir Kaya, Ayşegül Tel Kankılıç, Nur Ecer

**Affiliations:** ^1^ Department of Dermatology and Venereology Gazi Yaşargil Training and Research Hospital Diyarbakır Turkey; ^2^ Department of Dermatology and Venereology Istinye University Hospital Medical Park Gaziosmanpaşa Istanbul Turkey; ^3^ Department of Dermatology and Venereology Ergani State Hospital Diyarbakır Turkey; ^4^ Private Dermatology Practice Diyarbakır Turkey

**Keywords:** aesthetic dermatology, artificial intelligence, decision support, facial analysis, facial asymmetry, inter‐rater reliability, morphometric analysis, treatment planning

## Abstract

**Background:**

Facial aesthetic assessment is inherently subjective, leading to significant inter‐observer variability in treatment planning. Artificial intelligence (AI) offers a potential framework for standardized morphometric reporting.

**Objective:**

To evaluate whether AI‐augmented morphometric reports improve the consistency of simulated aesthetic treatment plans among dermatologists.

**Methods:**

Six dermatologists (3 experts, 3 residents) proposed treatment plans (neurotoxin units and filler volumes) for 76 standardized frontal images from the Chicago Face Database. In Phase I (Baseline), raters used visual intuition. In Phase II (AI‐Augmented), raters were provided with objective morphometric reports. The primary outcome was the change in inter‐rater reliability, measured by the Intraclass Correlation Coefficient (ICC(2,1)).

**Results:**

Significant baseline asymmetry (> 1.5 scaled units) was present in 58.4% of cases. The overall ICC(2,1) improved from 0.62 (95% CI: 0.54–0.69) to 0.88 (95% CI: 0.81–0.93) following AI augmentation. The most substantial improvement was observed in the resident group (ICC: 0.46 to 0.84). The mean absolute deviation from the expert‐group mean declined from 0.34 to 0.12 scaled units.

**Conclusion:**

AI‐augmented morphometric reporting was associated with improved inter‐rater agreement and reduced variability in simulated facial aesthetic treatment planning, particularly among less experienced raters. However, improved agreement does not necessarily reflect improved clinical accuracy, and future validation against clinical outcomes is warranted. The AI system functioned as a decision‐support tool rather than a replacement for clinician judgment.

## 1 Introduction

Facial aesthetic treatment planning remains one of the most subjective areas of dermatologic and cosmetic practice. Although clinicians routinely rely on structured facial analysis, treatment decisions in daily practice are still heavily influenced by individual visual judgment, professional experience, and personal aesthetic preference. This subjectivity may lead to meaningful inter‐physician variability in the selection of treatment zones, prioritization of asymmetries, and estimation of injectable doses. Even in procedures that are commonly standardized in principle, such as botulinum toxin or soft‐tissue filler applications, the assessment stage remains vulnerable to inconsistency because facial attractiveness, balance, and symmetry are interpreted through both biologic and culturally shaped perceptual frameworks. Studies evaluating facial aesthetic outcomes have also shown that aesthetic judgments may vary between observers, underscoring the limitations of purely intuitive evaluation [1].

Contemporary facial aesthetics has therefore moved beyond rigid neoclassical ideals and increasingly emphasizes individualized, diversity‐aware, and evidence‐based assessment. Recent literature highlights that modern facial evaluation should not rely solely on generalized ideals such as the golden ratio or classical proportions, but should also integrate symmetry, facial shape, expression, skin quality, and patient‐specific expectations within a broader decision‐making framework. In parallel, consensus‐oriented work on facial aesthetic assessment has reinforced that careful pretreatment evaluation is central to both treatment success and patient satisfaction. In this context, the need for more reproducible and objective assessment tools has become increasingly apparent, particularly in settings where clinicians with different levels of experience may interpret the same face differently [2].

Artificial intelligence (AI) and machine learning have emerged as promising tools to address this problem. A recent systematic review of AI and machine learning in facial aesthetic surgery identified 66 eligible studies and concluded that these technologies may improve diagnostic accuracy, predictive capabilities, patient counseling, and treatment planning. Similarly, recent reviews in aesthetic dermatology suggest that AI‐based systems are becoming increasingly relevant for image‐based analysis, treatment personalization, and clinical workflow support, while also emphasizing the need for validation, transparency, and careful interpretation. Thus, the most meaningful role of AI in facial aesthetics may not be to replace physician judgment, but rather to supplement it with objective morphometric information that reduces unwarranted variation in clinical decisions [3, 4].

Among currently available computer vision tools, facial landmarking systems offer a practical foundation for quantitative facial analysis. MediaPipe Face Mesh, for example, can estimate 468 three‐dimensional facial landmarks in real time from a single image without requiring a dedicated depth sensor. This type of landmark‐based framework is well suited to the analysis of facial proportions, midline deviation, contour asymmetry, and side‐to‐side differences that may be difficult to appreciate consistently with unaided visual inspection alone. However, despite growing technical interest in AI‐assisted facial analysis, evidence remains limited regarding whether these tools actually improve agreement among physicians during aesthetic treatment planning [5].

Standardized face databases may provide a useful experimental environment for addressing this gap. The Chicago Face Database (CFD) is a widely used resource consisting of high‐resolution, standardized facial photographs with extensive norming data, and version 2 has been further expanded with landmark templates for neutral faces. At the same time, morphometric studies comparing facial databases have shown that asymmetry‐related measurements may vary across datasets and populations, indicating that image‐based findings should be interpreted within their demographic and methodological boundaries. Against this background, the present study was designed to evaluate whether AI‐augmented morphometric reporting can improve inter‐physician agreement in facial aesthetic treatment planning using a multi‐rater assessment of standardized facial images. We further aimed to explore whether such augmentation may reduce variability between less experienced and more experienced evaluators [6, 7, 8].

## 2 Materials and Methods

### 2.1 Study Design and Ethics

This study was conducted as a two‐session, multi‐rater repeated‐measures experimental simulation. Since the study utilized a publicly available, de‐identified facial image database (Chicago Face Database v2.0) and involved no direct patient intervention or identifiable personal data, institutional review board (IRB) approval or formal exemption was obtained in accordance with local institutional requirements. The study procedures involving participating physicians were conducted in accordance with institutional ethical principles, and all raters provided written informed consent for the analysis of their clinical decision‐making data.

### 2.2 Participants and Standardized Instructions

Six physicians from the dermatology department participated as raters and were categorized into two experience‐based cohorts: an Expert Group (*n* = 3), consisting of board‐certified dermatologists with more than 10 years of clinical experience in aesthetic injectables, and a Resident Group (*n* = 3), consisting of dermatology residents with less than 3 years of specialized training. To ensure uniform dosing assumptions, all raters received a standardized written brief regarding general product conventions and simulated treatment guidelines, covering both neurotoxin concentrations and hyaluronic acid (HA) filler properties prior to the assessment phases. All participants were blinded to the specific study hypotheses.

### 2.3 Dataset and Internal Scaling

A total of 76 standardized, neutral‐expression frontal portraits (38 male, 38 female) were sampled from the Chicago Face Database (CFD) v2.0. Images were screened for high‐resolution clarity and consistent frontal orientation. To ensure comparability across the cohort, inter‐pupillary distance (IPD) was used as an internal scaling reference. All subsequent measurements were expressed in scaled units relative to the IPD to maintain consistency and reduce pixel‐to‐metric variability across images. The > 1.5 scaled‐unit threshold was defined a priori as an operational cutoff to distinguish more pronounced asymmetry from low‐grade variation within the study dataset, rather than as a formally outcome‐validated clinical threshold.

### 2.4 Automated Morphometric Pipeline

Facial analysis was performed using a reproducible open‐source computational pipeline. Dense facial landmarks were generated using MediaPipe Face Mesh (Google LLC), which provides 468 landmarks supplemented by peri‐iris coordinates for IPD‐based internal scaling. To reduce the influence of minor head tilt and non‐morphological positional variation, bilateral homologous landmark pairs from the periorbital, midfacial, and mandibular regions were reflected across the estimated mid‐sagittal plane and aligned using Procrustes superimposition.

Custom Python scripts using NumPy and Pandas were then used to compute morphometric indices. The Asymmetry Index (AIX) was calculated as the mean Euclidean distance across predefined bilateral landmark pairs after midline reflection and geometric alignment. In addition, vertical facial proportional estimates were derived from stable midline landmarks, specifically the glabella, nasion, subnasale, and gnathion, to improve robustness in two‐dimensional facial images while avoiding the variability associated with the hairline (trichion).

### 2.5 Experimental Protocol

The study utilized a two‐session design with a 3‐week washout period to mitigate recall bias. During Phase I (Baseline), raters performed visual assessments of raw CFD images and proposed a Simulated Treatment Plan, including botulinum toxin units for the glabella, frontalis, and orbicularis oculi regions and soft‐tissue filler volumes for the zygomatic arch, nasolabial fold, and mentum, yielding six predefined anatomical zones. In Phase II (AI‐Augmented), raters re‐evaluated the same 76 images in a randomized sequence. During this phase, each image was accompanied by a Standardized Morphometric Report detailing objective asymmetry scores (e.g., “Left malar projection is 2.1 scaled units lower than the right side”).

### 2.6 Statistical Analysis

Inter‐rater reliability was assessed as the primary outcome using the intraclass correlation coefficient (ICC). A two‐way random‐effects model, ICC(2,1), with absolute agreement and single‐measure estimation was selected to account for both rater‐ and case‐related variance. As secondary outcomes, the mean absolute deviation from the expert‐group mean and the coefficient of variation (CV) were calculated for each phase to assess decision convergence. Bland–Altman plots were generated as supplementary visualizations to examine within‐case differences in proposed treatment doses between Phase I and Phase II. Differences in ICC estimates between phases were evaluated using bootstrap‐based 95% confidence intervals (CIs). Statistical analyses were performed using SPSS version 28.0 and R version 4.2.2, with statistical significance set at *p* < 0.05.

## 3 Results

### 3.1 Prevalence of Baseline Morphometric Asymmetry

Automated analysis of the 76‐case image cohort identified a spectrum of morphometric asymmetries. Using a pre‐defined threshold of > 1.5 scaled units relative to the IPD—selected a priori to distinguish more pronounced asymmetry from minor positional variation—significant deviation was identified in 58.4% of the images. The distribution of these findings varied by anatomical region. The highest prevalence of asymmetry was observed in vertical brow positioning (61.8%), followed by midfacial lateral (malar) asymmetry (48.7%) and menton midline deviation (45.3%). Table 1 summarizes these objective baseline deviations, which served as the reference data for the subsequent AI‐augmented assessment phase.

**TABLE 1:** Distribution of AI‐detected morphometric deviations (*N* = 76 cases).

Anatomical region	Mean deviation (scaled units)	Frequency of deviation (> 1.5 units)
Upper face (brow height)	1.9 ± 0.7	61.8%
Mid‐face (malar projection)	1.8 ± 0.5	48.7%
Lower face (menton deviation)	2.3 ± 1.1	45.3%
Combined asymmetry index	2.1 ± 0.8	58.4%

### 3.2 Primary Outcome: Inter‐Rater Reliability

Inter‐rater agreement showed a measurable upward shift following the introduction of the morphometric reports. In Phase I (Baseline), the overall ICC(2,1) for simulated filler volumes and toxin units was 0.62 (95% CI: 0.54–0.69), reflecting moderate agreement among the six raters. In Phase II (AI‐Augmented), the overall ICC(2,1) increased to 0.88 (95% CI: 0.81–0.93). This improvement was supported by bootstrap‐based 95% confidence intervals showing clear separation between the two phases. Notably, the Resident Group demonstrated the most substantial shift, with their ICC(2,1) increasing from 0.46 (95% CI: 0.38–0.55) to 0.84 (95% CI: 0.78–0.89), indicating substantially improved within‐group agreement during the AI‐augmented phase (Table 2).

**TABLE 2:** Comparison of inter‐rater reliability [ICC(2,1)] between phases.

Rater group	Phase I (baseline)	Phase II (AI‐augmented)	ΔICC
Expert group (*n* = 3)	0.71 (0.64–0.78)	0.90 (0.86–0.93)	+0.19
Resident group (*n* = 3)	0.46 (0.38–0.55)	0.84 (0.78–0.89)	+0.38
Total (*N* = 6)	0.62 (0.54–0.69)	0.88 (0.81–0.93)	+0.26

### 3.3 Secondary Outcomes: Decision Convergence and Revision Rates

The introduction of objective morphometric data was associated with greater convergence in treatment recommendations. The mean absolute deviation (MAD) from the expert‐group mean declined markedly, dropping from an overall average of 0.34 scaled units in Phase I to 0.12 scaled units in Phase II. Similarly, the coefficient of variation (CV) decreased across all treatment zones, with the most illustrative reduction in dispersion observed for malar filler recommendations, where the Bland–Altman limits of agreement (LoA) narrowed from ±0.76 mL in Phase I to ±0.24 mL in Phase II. Raters modified their initial treatment plans in 71.1% of cases—defined as a change of ≥ 1 toxin unit or ≥ 0.1 mL filler in at least one anatomical zone—after viewing the AI reports. These findings were consistent with greater convergence in treatment recommendations during the AI‐augmented phase.

## 4 Discussion

The present study suggests that AI‐augmented morphometric reporting may improve consistency in image‐based facial aesthetic treatment planning among dermatology raters. The principal finding was a marked increase in inter‐rater reliability after the introduction of standardized morphometric reports, accompanied by reduced dispersion in treatment recommendations. Taken together, these findings support the view that AI may function less as a replacement for clinical judgment and more as a structured decision‐support tool that mitigates inter‐observer drift and reduces subjective assessment bias during facial evaluation. This interpretation is consistent with prior literature showing that facial aesthetic assessment is inherently vulnerable to observer‐dependent variability and that more structured assessment frameworks are needed in routine practice [1, 2]. It also aligns with recent reviews describing AI as a potentially valuable adjunct in facial aesthetics, particularly when used to improve precision, reproducibility, and treatment planning rather than to automate decisions outright [3, 4].

A clinically relevant aspect of our findings is that the largest relative gain in agreement was observed in the resident group. Although this subgroup was small, this pattern suggests that objective morphometric feedback may reduce inter‐observer drift across different levels of clinical experience by providing a shared analytical reference during treatment planning. In aesthetic medicine, where even subtle differences in zone prioritization or dose selection may influence outcomes and patient satisfaction, greater consistency may have practical value not only for education but also for institutional standardization and patient safety. When clinicians at different levels of training interpret facial proportions through a more common analytic framework, treatment planning may become more transparent, reproducible, and less dependent on individual intuition alone. Prior validation studies of facial photonumeric scales have similarly shown that standardized rating systems can improve inter‐rater reliability when applied by trained clinicians [9]. Our findings extend that principle from ordinal aesthetic grading toward AI‐assisted, geometry‐based simulated treatment planning, suggesting that landmark‐derived reports may serve as an additional layer of standardization and facilitate clinical decision harmonization between junior and senior practitioners. This interpretation is also compatible with recent discussions of AI‐supported workflow standardization in cosmetic dermatology [10].

An important interpretive distinction, however, is that improved agreement should not automatically be equated with improved clinical accuracy. The present study was designed to assess convergence in simulated treatment planning under standardized conditions rather than clinical accuracy against an external gold standard. In the absence of such a gold standard, the findings reflect inter‐rater agreement rather than clinical validity. Thus, the observed reduction in inter‐observer drift is best interpreted as evidence that objective landmark‐derived reporting may rationalize and stabilize decision‐making, not that AI‐assisted recommendations are inherently more correct in all circumstances. At the same time, unlike purely opinion‐based consensus, the convergence observed here was anchored to objective anatomic landmarks and quantified morphometric relationships. In that sense, AI may help reduce error not by replacing judgment, but by constraining judgment within a more reproducible anatomical framework. This distinction between agreement and correctness is particularly important in aesthetic medicine, where the appropriateness of a treatment plan remains dependent on clinical context, patient preference, and procedural judgment, and where high inter‐rater reliability does not necessarily equate to optimal clinical outcomes [2, 3, 4].

The baseline morphometric findings also warrant comment. More than half of the standardized images demonstrated measurable asymmetry above the pre‐defined threshold, with the greatest prevalence observed in brow position and lower‐face deviation. This is not unexpected, as minor facial asymmetry is common in the general population, whereas more pronounced asymmetry may more strongly influence aesthetic perception and treatment choices [11]. From a practical standpoint, the value of AI in this setting may lie not merely in detecting asymmetry, but in translating subtle side‐to‐side variation into a consistent and quantifiable format. This may help clinicians distinguish more meaningful deviation from low‐grade normal asymmetry, thereby reducing both overcorrection and under‐recognition. Recent reviews in aesthetic dermatology have emphasized that AI‐based systems may be particularly useful when subtle image‐derived findings must be incorporated into individualized yet reproducible treatment plans [4, 10].

The asymmetry threshold used in the present study also deserves clarification. The > 1.5 scaled‐unit cutoff was defined a priori as an operational threshold intended to separate more pronounced morphometric deviation from low‐grade variation within the study dataset, rather than as a formally outcome‐validated clinical threshold. Although this approach was methodologically useful for standardizing baseline categorization, its direct visual or clinical detectability should be interpreted with caution. Future studies should evaluate whether similar thresholds correspond to clinician‐recognizable asymmetry, patient‐reported concern, or measurable differences in post‐treatment satisfaction.

Despite these considerations, the findings support a meaningful role for AI‐assisted morphometric analysis in aesthetic dermatology. Rather than replacing the clinician's aesthetic judgment, structured AI reports may help anchor that judgment within a reproducible geometric framework. This may be particularly useful in educational settings, multiclinician practices, and research protocols where standardization is essential. In that sense, the most important contribution of AI may not be the automation of aesthetic intuition, but the mitigation of unwarranted variability in how facial features are interpreted. By providing a reproducible geometric anchor, AI‐augmented reporting may facilitate a more transparent and standardized transition from visual diagnosis to simulated procedural planning.

## 5 Limitations

Several limitations of the present study should be acknowledged. First, the study was designed as an experimental simulation based on static frontal facial photographs rather than real patient consultations. Accordingly, the observed improvement reflects greater consistency in simulated treatment planning rather than demonstrated superiority in clinical outcomes. In routine practice, aesthetic decision‐making is also influenced by dynamic facial movement, profile analysis, tissue quality, age‐related volumetric change, patient preference, and safety considerations, none of which can be fully captured in a static 2D image.

Second, the use of static 2D frontal images significantly limits the validity of simulated treatment planning, particularly for volumetric assessments such as filler injections. Although 2D images offer practical standardization for high‐throughput analysis, this format has inherent constraints. Projection, contour transition, and volumetric balance are fundamentally three‐dimensional features and may not be fully represented in static 2D facial analysis. Dynamic facial expressions may also accentuate underlying skeletal or muscular asymmetries that remain less apparent on frontal static images. This limitation is particularly critical for filler planning, where accurate volumetric assessment requires three‐dimensional spatial information that cannot be reliably inferred from a single frontal view. Therefore, the present findings should be interpreted as a baseline validation of AI‐assisted planning rather than a comprehensive representation of real‐world facial assessment. Prior work comparing 2D and 3D facial analyses has shown that 3D approaches may provide superior reliability for selected facial and volumetric assessments, which are particularly relevant to filler planning [12].

Third, the small number of raters (*n* = 6) reduces the robustness of the ICC estimates and limits generalizability, especially for subgroup analyses. The relatively small rater panel may have limited not only the precision of subgroup ICC(2,1) estimates but also the breadth of aesthetic perspectives represented in the study. With only three raters per experience group, the confidence intervals for subgroup‐specific reliability estimates are necessarily wide, and the findings should be interpreted with appropriate caution. This is especially relevant in facial aesthetics, where treatment preferences may vary according to training background, clinical philosophy, and sociocultural context. Future studies should therefore include larger, multi‐institutional, and ideally international clinician panels to improve external validity and provide more robust ICC estimates.

Fourth, the asymmetry threshold (> 1.5 scaled units) is arbitrary and not clinically validated, which may impact interpretation of the findings. Although the > 1.5 scaled‐unit threshold was useful for operationally distinguishing more pronounced morphometric deviation from low‐grade variation within the study dataset, it was defined a priori as an operational cutoff rather than a formally outcome‐validated clinical threshold. Its direct visual detectability and clinical interpretability therefore remain uncertain. Further studies are needed to determine whether comparable thresholds correspond to clinician‐recognizable asymmetry, patient‐reported concern, or measurable differences in post‐treatment satisfaction.

Finally, although the Chicago Face Database is a valuable research resource because it provides high‐resolution, standardized facial images with accompanying norming data [6, 7], morphometric studies have shown that facial measurements and asymmetry‐related indices may vary across datasets and population groups [8]. Accordingly, the present threshold definitions and morphometric interpretations should not be assumed to carry identical aesthetic weight across all ethnic backgrounds or facial phenotypes. In particular, canonical proportional frameworks derived from neoclassical ideals may require cautious interpretation when applied across non‐Caucasian facial types. In this context, AI should be regarded as a tool that generates structured numeric descriptions, whereas the clinician remains responsible for interpreting those findings in light of the patient's ethnic background, facial identity, and aesthetic goals.

## 6 Conclusion

The present study suggests that AI‐augmented morphometric reporting may improve the consistency of image‐based facial aesthetic treatment planning by providing a reproducible geometric anchor for clinical assessment. Objective landmark‐derived data appeared to reduce inter‐observer drift and support greater convergence in treatment recommendations, particularly among less experienced raters. In this respect, AI‐supported workflows may contribute to a more transparent and standardized transition from visual assessment to simulated procedural planning.

Rather than replacing clinician expertise, AI may function as a practical decision‐support tool that strengthens the reproducibility of facial analysis while reducing subjective assessment bias. As aesthetic medicine increasingly emphasizes precision, standardization, and quality assurance, structured morphometric feedback may offer value in both clinical education and treatment planning. Future research integrating three‐dimensional imaging, dynamic facial analysis, larger rater panels, and real‐world longitudinal outcomes will be necessary to determine whether improved decision consistency translates into better patient‐centered aesthetic outcomes.

## Author Contributions

Ömer Karakoyun contributed to the conception and design of the study, supervised the project, interpreted the findings, and drafted the manuscript. Kadir Kaya contributed to data curation, formal analysis, software support, and visualization. Ayşegül Tel Kankılıç contributed to the literature review, investigation, and manuscript preparation. Nur Ecer contributed to critical revision of the manuscript for important intellectual content and final review. All authors read and approved the final manuscript.

## Funding

The authors have nothing to report.

## Ethics Statement

This study was based exclusively on a publicly available, de‐identified facial image dataset and did not involve direct patient contact, clinical intervention, or identifiable personal health information. Therefore, formal ethics committee approval and informed consent from image subjects were not required. The decision‐making data of participating physician raters was collected and analyzed in anonymized form.

## Consent

The authors have nothing to report.

## Conflicts of Interest

The authors declare no conflicts of interest.

## Data Availability

The facial image dataset used in this study was obtained from the publicly available Chicago Face Database (CFD) v2.0, subject to the terms and conditions of the database. Derived study materials and analytic outputs are available from the corresponding author on reasonable request.
